# Patient Retention and Adherence to Antiretrovirals in a Large Antiretroviral Therapy Program in Nigeria: A Longitudinal Analysis for Risk Factors

**DOI:** 10.1371/journal.pone.0010584

**Published:** 2010-05-11

**Authors:** Man Charurat, Modupe Oyegunle, Renata Benjamin, Abdulrazaq Habib, Emeka Eze, Prince Ele, Iquo Ibanga, Samuel Ajayi, Maria Eng, Prosanta Mondal, Usman Gebi, Emilia Iwu, Mary-Ann Etiebet, Alash'le Abimiku, Patrick Dakum, John Farley, William Blattner

**Affiliations:** 1 Institute of Human Virology, University of Maryland School of Medicine, Baltimore, Maryland, United States of America; 2 Aminu Kano Teaching Hospital, Kano, Kano State, Nigeria; 3 University of Benin Teaching Hospital, Benin, Edo State, Nigeria; 4 Nnamdi Azikwe University Teaching Hospital, Nnewi, Anambra State, Nigeria; 5 University of Calabar Teaching Hospital, Calabar, Cross River State, Nigeria; 6 University of Abuja Teaching Hospital, Abuja, Federal Capital Territory, Nigeria; 7 Institute of Human Virology Nigeria, Abuja, Federal Capital Territory, Nigeria; 8 Department of Family and Community Health, University of Maryland School of Nursing, Baltimore, Maryland, United States of America; University of Cape Town, South Africa

## Abstract

**Background:**

Substantial resources and patient commitment are required to successfully scale-up antiretroviral therapy (ART) and provide appropriate HIV management in resource-limited settings. We used pharmacy refill records to evaluate risk factors for loss to follow-up (LTFU) and non-adherence to ART in a large treatment cohort in Nigeria.

**Methods and Findings:**

We reviewed clinic records of adult patients initiating ART between March 2005 and July 2006 at five health facilities. Patients were classified as LTFU if they did not return >60 days from their expected visit. Pharmacy refill rates were calculated and used to assess non-adherence. We identified risk factors associated with LTFU and non-adherence using Cox and Generalized Estimating Equation (GEE) regressions, respectively. Of 5,760 patients initiating ART, 26% were LTFU. Female gender (p<0.001), post-secondary education (p = 0.03), and initiating treatment with zidovudine-containing (p = 0.004) or tenofovir-containing (p = 0.05) regimens were associated with decreased risk of LTFU, while patients with only primary education (p = 0.02) and those with baseline CD4 counts (cell/ml^3^) >350 and <100 were at a higher risk of LTFU compared to patients with baseline CD4 counts of 100–200. The adjusted GEE analysis showed that patients aged <35 years (p = 0.005), who traveled for >2 hours to the clinic (p = 0.03), had total ART duration of >6 months (p<0.001), and CD4 counts >200 at ART initiation were at a higher risk of non-adherence. Patients who disclosed their HIV status to spouse/family (p = 0.01) and were treated with tenofovir-containing regimens (p≤0.001) were more likely to be adherent.

**Conclusions:**

These findings formed the basis for implementing multiple pre-treatment visit preparation that promote disclosure and active community outreaching to support retention and adherence. Expansion of treatment access points of care to communities to diminish travel time may have a positive impact on adherence.

## Introduction

Nigeria is home to the second highest number of people living with HIV in the world and the highest in West Africa, with an estimated 2.6 million infected at the end of 2007[1]. Substantial resources and patient commitment are both required to successfully scale-up antiretroviral therapy (ART), minimize loss to follow-up (LTFU), and ensure optimal adherence in this setting.

Although substantial rates of LTFU have been shown in ART programs in resource limited settings[2], [3], [4], [5], ranging from 16%[6] to as high as 40% after 2 years[4], risk factors and patient-level and program-level characteristics of LTFU in these settings have received little attention because of limited access to longitudinal patient-level data. This is further compounded by the lack of mandate by donor agencies to report program metrics on patients' retention and patients' adherence to treatment[7].

Several studies in Africa have shown a high level of medication adherence[8] demonstrating that scale-up of ART in resource-limited settings can achieve durable immunologic and clinical outcomes. The majority of these findings were based on small study populations[2], [9], [10], supervised cohort[11], and/or patient-reported adherence level[10], [12]. Subsequent reports have raised concern regarding sub-optimal adherence when assessed by pill counts, pharmacy records, or electronic monitoring[13], [14], [15], [16], [17].

Lack of adherence to antiretroviral medications and attrition from health services contribute to poorer health outcomes and waste limited resources. Identifying patient characteristics that are associated with these outcomes could be used for making evidence-informed decisions to improve patient care and programmatic outcomes. In this paper, we describe risk factors for LTFU and non-adherence to antiretrovirals in a large antiretroviral therapy program in Nigeria.

## Methods

In 2005, the AIDS Care and Treatment in Nigeria (ACTION) project was created as a joint initiative between the Institute of Human Virology of the University of Maryland School of Medicine, the Institute of Human Virology Nigeria, the Nigerian Federal Ministry of Health, and local partner treatment facilities. The goals of ACTION are to implement a multidisciplinary program of primary HIV prevention, and HIV care and support that employs an evidence-based approach using robust strategic information systems to monitor performance and evaluate quality in order to continually improve program sustainability.

This is a retrospective analysis of existing data and was determined to be exempt from oversight after review by the National Health Research Ethics Committee of Nigeria and the University of Maryland Baltimore Institutional Review Board. Patients were included in the analysis if they were non-pregnant treatment-naive adults initiating first-line ART between March 2005 and July 2006 at five tertiary hospitals in Nigeria. Among the first in Nigeria to provide large-scale ART access through support from PEPFAR, the local partner treatment facilities included were the University of Abuja Teaching Hospital (UATH), the University of Benin Teaching Hospital (UBTH), the Aminu Kano Teaching Hospital (AKTH), the Nnamdi Azikwe University Teaching Hospital (NAUTH), and the University of Calabar Teaching Hospital (UCTH). All WHO clinical stage 4 patients, stage 3 patients with a CD4 cell count <350 cells/ul, and stage 1 and stage 2 patients with a CD4 cell count <200 ul were eligible for ART in accordance with the Nigerian National Guidelines[18]. Patients were followed from the time of ART initiation until either LTFU or to October 31, 2006 to allow for a minimum of 90 days in the interval of analysis.

### Clinical procedures and Data collection

At clinic enrollment, patients underwent a history and physical examination and were interviewed by staff in order to complete an intake record which included demographic data. During this period, adherence counseling was routinely provided to patients, but a regimented intensive HIV treatment preparation program was not offered as part of routine clinical care. Six first-line treatment regimens were prescribed: (stavudine [d4T] + lamivudine [3TC] + nevirapine [NVP]/efavirenz[EFV]), (zidovudine [ZDV] + 3TC + NVP/EFV), and (tenofovir [TDF] + 3TC + NVP/EFV). Although physicians were trained to prescribe ZDV as a first choice, with TDF used if anemia was present and utilize EFV with concomitant anti-tuberculosis treatment with exception for child-bearing age women, choice of regimen was at the discretion of the physician. Patients were scheduled for a follow-up visit one month after ART was first dispensed and then every two or three months at the discretion of the physician. A sufficient supply of ART was dispensed to last until the next scheduled visit. At each follow-up visit, patients underwent a history and physical and any side effects reported were recorded by the physician on a clinical encounter form. A pharmacy order form, which includes the date ART was dispensed, the formulations dispensed, and the quantity of each formulation dispensed, was completed by the physician at each visit and presented to the pharmacist for ART dispensing.

Form data were entered in real-time into the CAREWare health management information system. Originally developed for the US domestic HIV treatment and care program under the Ryan White CARE Act, the system has been adapted by the US Department of Health and Human Services Health Resources and Service Administration for resource-limited setting through PEPFAR and was deployed by the ACTION project.

### Statistical Analysis

For the LTFU analysis, time-to-event analytical method was used. Patients were considered LTFU when they did not return to the clinic more than 60 days from their last expected visit. The risk of LTFU was determined using Cox proportional hazard regression with ART initiation as time zero. The proportional hazards assumption was assessed with log-log plots and regression of the Schoenfeld residuals[19]; the goodness of fit was assessed by log-likelihood test.

For the non-adherence to ARV analysis, pharmacy dispensing records over the duration of ARV therapy for each patient were reviewed. Each patient was required to have been receiving ART for a minimum of 90 days to be included in the analysis. Days of medication dispensed between visits was calculated. A pharmacy refill adherence rate (Rx) was then calculated (days of medication dispensed/days between visits multiplied by 100). For each visit interval when the Rx is less than 95% (i.e. an episode of Rx<95%), a binary outcome of 1 was designated (i.e. a value of 0 denoted a visit where Rx ≥95%). Given the longitudinal nature of the outcome, the risk factor analysis for Rx<95% within the first 12 months of initiating ART were modeled as the odds of having Rx<95% at any given visit using Generalized Estimating Equation[20], [21] accounting for correlation between repeated measurements using a covariance structure of unequally spaced data. The quasi-likelihood information criterion (QIC) statistics was used to evaluate fit[22]. In addition to QIC, goodness of model fit was evaluated using Horton et al.′s goodness-of-fit for logistic regression estimated by GEE[23].

In both of these primary regression analyses of LTFU and non-adherence to ARV, potential covariates were first examined in unadjusted models. Factors associated at the p<0.05 level with the outcome and known risk factors for LTFU and non-adherence regardless of their level of significance were then evaluated in multivariate models. To control for confounding, variables that altered any significant relative hazards or odds ratios by ≥20% were retained. To account for missing data that may have not occurred at random, multiple imputations for missing data were performed using Markov chain Monte Carlo method (SAS Proc MI). The analyses of the complete-data obtained through the imputation did not change the results; therefore, only the primary regression results are shown.

In order to model the effect of time since ART initiation as a continuous risk factor on the outcome of Rx<95%, we fitted a cubic polynomial model using the GEE method to obtain the predicted values of the proportion of individuals with the outcome from the associated β coefficients. Similar to the procedure used in the multivariate analyses above, the model with the smallest QIC value was chosen as the best model of time since ART initiation.

Statistical analyses were performed using STATA 10.0 (College Station, TX) for the LTFU analysis and SAS 9.2 (Cary, NC) for the GEE analysis.

## Results

### Description of Study Population

The study population included 5,760 patients; 2,385 (59%) were female and the median age at time of enrollment was 35 years (25^th^–75^th^ percentile: 29–41 years) – **Table 1**. The median CD4 absolute count at ART initiation was 121 cells/mm^3^ with 76% of the patients having had CD4≤200 cells/mm^3^. The majority of the patients (51%) lived within 1 hour of travel time to the clinic. At the time of this analysis, the median time on ART was 215 days (25^th^–75^th^ percentile: 110–361 days) with 44% of patients on ART for less than or equal to 6 months, 32% on ART between 6 and 12 months, and 24% on ART for between 12 and 18 months. There were a total of 44,571 person-months of follow-up. The majority of the patients were initiated on zidovudine-containing or stavudine-containing first-line regimens (46% and 41%, respectively). The patient volume was highest at the University of Abuja Teaching Hospital (n = 1,829 patients) and lowest at the University of Calabar Teaching Hospital (n = 398 patients).

**Table 1:** Characteristics of the Study Population (N = 5760).

Characteristics		Number (%)
**Gender**	Female	3375 (59)
	Male	2385 (41)
**Age (years)**	≤25	683 (12)
	25–30	1262 (22)
	31–35	1246 (22)
	35–40	1071 (19)
	41–45	850 (15)
	46+	648 (11)
**Health Facilities**	University of Abuja Teaching Hospital	1829 (32)
	University of Benin Teaching Hospital	1140 (20)
	Aminu Kano Teaching Hospital	989 (17)
	Nnmadi Azikwe University Teaching Hospital	1404 (24)
	University of Calabar Teaching Hospital	398 (7)
**Educational Level**	Post-Secondary	1239 (22)
	Completed Secondary	2049 (37)
	Completed Primary	1263 (23)
	Quranic education	260 (5)
	None or some Primary	531 (9)
	Others	163 (3)
	(Missing)	255
**Marital Status**	Currently Married	3134 (55)
	Currently Not Married	2596 (45)
	(Missing)	30
**Employment Status**	Student or Unemployed	1146 (22)
	Other/Retired	2224 (42)
	Employed	1942 (37)
	(Missing)	448
**Time to Travel to the ART Center**	≤1 Hour	2862 (51)
	>1–2 Hours	1533 (27)
	>2 Hours	1196 (21)
	(Missing)	169
**CD4 Absolute Count at Initiation of ART**	Median (Interquartile Range)	121(141)
	>350 cells/mL^3^	251 (5)
	201–350 cells/mL^3^	858 (19)
	100–200 cells/mL^3^	1567 (34)
	<100 cells/mL^3^	1954 (42)
	(Missing)	1130
**First Line ART Regimen Prescribed at Baseline**	ZDV/3TC/NVP or EFV	2669 (46)
	TDF/3TC/NVP or EFV	731 (13)
	d4T/3TC/NVP or EFV	2360 (41)
**ART Duration**	≤6 Months	2529 (44)
	>6 and <12 Months	1826 (32)
	12–18 Months	1405 (24)

### Risk Factors of Loss to Follow-up

**Figure 1:**
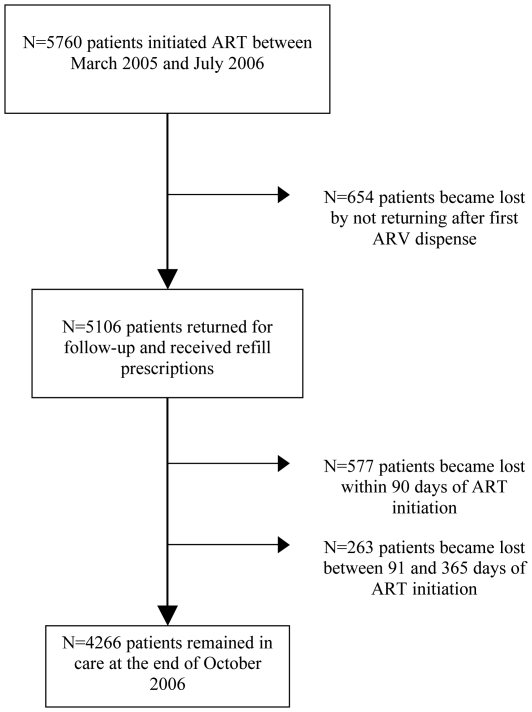
Study Population of Patients Initiating ART between March 2005 and July 2006.

During the follow-up period, 1494 (25.9%) patients became LTFU (**Figure 1**). Cumulative incidence of LTFU was 22.9% at 6 months and 25.3% at 12 months. Of 1494 patients identified as lost, 654 (43.8%) patients never returned for further visits after the first clinic visit when ARVs were dispensed. Of 840 patients who were lost later in the course of follow-up, 577 (68.7%)were lost in the first90 days after ART initiation. LTFU was highest at the Nnamdi Azikwe University Teaching Hospital (32.5%) and lowest at the University of Benin Teaching Hospital (22.0%).

The characteristics associated with LTFU are shown in **Table 2**. Female patients were less likely to become LTFU (HR = 0.75, 95%CI: 0.67–0.82). Patients with post-secondary education (HR = 0.80, 95%CI: 0.65–0.99) or other unspecified education (HR = 0.61, 95%CI: 0.41–0.93) were less likely to become LTFU compared to individuals with no primary or some primary education. Patients in the highest CD4 cells count category (>350 cells/mm^3^) and those in the lowest category (<100 cells/mm^3^) at ART initiation had higher risk of LTFU (HR = 1.47 and HR = 1.41, respectively) relative to patients with CD4 between 100 and 200 cells/mm^3^. Use of a ZDV-containing first-line regimen was associated with decreased risk of LTFU compared with a d4T-containing regimen (HR0.85, 95%CI: 0.76–0.95). The findings, specifically the increased risk of LTFU associated with being male, CD4 at initiation of ART <100 and >350, and with d4T-containing first-line regimen, remain significant when the analyses were stratified by site (data not shown). There was no difference in LTFU risk between EFV-containing and NVP-containing regimens (26.2% vs. 25.9%, p = 0.84). In the adjusted Cox Regression analysis of 4,177 (72.5%) patients with complete data variables, risk of LTFU remained associated with gender, educational level, CD4 at the time of ART initiation and types of first-line regimen. After adjusting for these covariates, younger age at clinic enrollment was associated with increased risk of LTFU.

**Table 2:** Cox Regression Analyses of Characteristics Associated with Loss to Follow-Up (LTFU) Among Treatment-Naïve Adult Nigerian Patients.

		Total	LTFU, n = 1494, (26%)		Unadjusted		Adjusted^b^	
		N^a^	n	%	HR (95% CI)	P-value	HR (95% CI)	P-value
**Gender**	Female	3375	773	23	0.75(0.67–0.82)	**<0.001**	0.70 (0.62–0.81)	**<0.001**
	Male	2385	721	30	Ref		Ref	
**Age (years)**	≤25	683	167	24	1.01 (0.84–1.26)	0.90	1.36 (1.03–1.79)	**0.03**
	25–30	1262	329	26	1.09 (0.90–1.32)	0.37	1.31 (1.03–1.65)	**0.03**
	31–35	1246	333	27	1.11 (0.92–1.35)	0.27	1.19 (0.94–1.50)	0.15
	35–40	1071	303	28	1.18 (0.97–1.45)	0.09	1.30 (1.03–1.64)	**0.03**
	41–45	850	209	25	1.03 (0.83–1.27)	0.76	1.00 (0.78–1.29)	0.97
	46+	648	153	24	Ref		Ref	
**Educational Level**	Post-Secondary	1239	271	22	0.80 (0.65–0.99)	**0.03**	0.75 (0.58–0.96)	**0.03**
	Completed Secondary	2049	564	28	1.02 (0.85–1.23)	0.82	0.97 (0.78–1.21)	0.67
	Completed Primary	1263	362	29	1.07 (0.88–1.30)	0.50	1.01 (0.80–1.27)	0.93
	Quranic education	260	75	29	1.02 (0.77–1.35)	0.87	1.08 (0.74–1.56)	0.70
	Others	163	27	17	0.61 (0.41–0.93)	**0.02**	0.54 (0.33–0.89)	**0.02**
	None or some Primary	531	143	27	Ref		Ref	
**Marital Status**	Currently Married	3134	819	26	1.01 (0.92–1.12)	0.78	0.93 (0.82–1.06)	0.30
	Currently Not Married	2596	666	26	Ref		Ref	
**Employment Status**	Student or Unemployed	1146	269	23	0.90 (0.78–1.04)	0.17	0.99 (0.82–1.20)	0.97
	Other/Retired	2224	614	28	1.04 (0.93–1.18)	0.45	1.04 (0.90–1.20)	0.53
	Employed	1942	511	26	Ref		Ref	
**Disclosure of HIV+ Status**	To Spouse or Family Members	4058	1000	25	0.96 (0.82–1.12)	0.57		
	To Others Only	240	55	23	0.89 (0.66–1.20)	0.45		
	To No One	733	187	26	Ref			
**Time to Travel to the ART**	>2 Hours	1196	290	24	0.90 (0.78–1.02)	0.11		
**Center**	>1–2 Hours	1533	400	26	0.97(0.86–1.10)	0.68		
	≤1 Hour	2862	768	27	Ref			
**CD4 Absolute Count at**	>350 cells/mL^3^	251	78	31	1.47 (1.15–.1.89)	**0.002**	1.62 (1.25–2.11)	**<0.001**
**Initiation of ART**	201–350 cells/mL^3^	858	187	22	1.04 (0.87–1.25)	0.63	1.07 (0.89–1.30)	0.47
	100–200 cells/mL^3^	1567	329	21	Ref		Ref	
	<100 cells/mL^3^	1954	576	30	1.41 (1.23–1.61)	**<0.001**	1.37 (1.18–1.57)	**<0.001**
**First Line ART Regimen**	ZDV+3TC+NVP or EFV	2669	630	24	0.85 (0.76–0.95)	**0.004**	0.76 (0.67–0.87)	**<0.001**
**Prescribed**	TDF+3TC+NVP or EFV	731	196	27	0.93 (0.79–1.09)	0.37	0.83 (0.69–1.00)	**0.05**
	d4T+3TC+NVP or EFV	2360	668	28	Ref		Ref	

a Totals always do not sum to 5,760 due to missing data;

b A total of 4,177 subjects included in the adjusted model; HR, hazard ratio; CI, confidence interval.

### Risk Factors of Non-Adherence to Antiretrovirals

For the analysis of pharmacy refill rates, 4529 patients who returned for further visits beyond 90 days of ART initiation were included. Of these, 3362 (74.2%) had a pharmacy refill rate <95% at any time during follow-up. A total of 1747 (38.5%) had a summary pharmacy refill at the last visit <95%; of which 15.8% had Rx<50%, 33.6% with Rx of 50–79%, and 50.6% with Rx of 80–95%. In the bivariate analyses of those characteristics associated with pharmacy refill rate <95% (**Table 3**), younger age groups (vs. patients over 45 years of age), Quranic education (vs. having had none or some primary school), unemployment (vs. being employed), and a CD4 count of 200 or more at time of ART initiation were associated with increased odds of Rx<95%. Having disclosed HIV status to either spouse or family members was associated with decreased odds of Rx<95% (OR = 0.85, 95CI: 0.76–0.94) compared to those who did not disclose to anyone. Patients initiated on a ZDV-containing or TDF-containing first-line regimen were less likely to have Rx<95% compared to patients initiated on a D4T-based regimen. There was no difference in the risk of Rx<95% between EFV-containing and NVP-containing regimens (data not shown).

**Table 3:** Associations with Pharmacy Refill (Rx) <95% Among Treatment-Naïve Adult Nigerian Patients based on GEE.

			Unadjusted		Adjusted^b^	
		N^a^	OR (95% CI)	P-value	OR (95% CI)	P-value
**Gender**	Female	2743	1.05 (0.98–1.12)	0.15	0.99 (0.91–1.09)	0.87
	Male	1786	Ref		Ref	
**Age (years)**	≤25	551	1.22 (1.07–1.38)	**0.003**	1.18 (0.98–1.41)	0.07
	26–30	979	1.29 (1.15–1.44)	**<0.0001**	1.28 (1.10–1.48)	**0.001**
	31–35	971	1.15 (1.02–1.28)	**0.02**	1.16 (1.00–1.34)	**0.044**
	36–40	823	1.18 (1.04–1.32)	**0.008**	1.12 (0.97–1.30)	0.12
	41–45	603	1.02 (0.89–1.16)	0.80	1.03 (0.88–1.21)	0.70
	≥46	602	Ref		Ref	
**Educational Level**	Post-Secondary	1011	1.05 (0.92–1.19)	0.46	1.03 (0.88–1.21)	0.73
	Completed Secondary	1588	1.03 (0.91–1.16)	0.69	1.02 (0.89–1.18)	0.75
	Completed Primary	962	0.94 (0.82–1.07)	0.34	0.98 (0.84–1.14)	0.79
	Quranic	205	1.40 (1.15–1.69)	**0.0006**	1.37 (1.01–1.84)	**0.040**
	None or Some Primary	414	Ref		Ref	
**Marital Status**	Currently Married	2454	0.96 (0.90–1.02)	0.20		
	Currently Not Married	2052	Ref			
**Disclosure of HIV+ Status**	To Spouse or Family Members	3228	0.85 (0.76–0.94)	**0.002**	0.85 (0.75–0.97)	**0.01**
	To Others Only	193	0.94 (0.78–1.12)	0.49	0.92 (0.74–1.14)	0.45
	To No One	579	Ref		Ref	
**Employment Status**	Student or Unemployed	931	1.16 (1.06–1.27)	**0.001**	1.10 (0.98–1.24)	0.12
	Other/Retired	1705	0.99 (0.91–1.07)	0.76	1.07 (0.98–1.18)	0.14
	Employed	1513	Ref		Ref	
**Time to Travel to the ART Center**	>2 Hours	963	1.01 (0.93–1.10)	0.80	1.11 (1.01–1.23)	**0.039**
	>1–2 Hours	1202	0.97 (0.89–1.05)	0.39	1.07 (0.97–1.17)	0.19
	≤1 Hour	2223	Ref		Ref	
**CD4 Absolute Count at Initiation of ART**	>350 Cells/mL^3^	187	1.38 (1.18–1.62)	**<0.0001**	1.25 (1.05–1.49)	**0.01**
	>200 and ≤350 Cells/mL^3^	707	1.20 (1.08–1.32)	**0.0006**	1.18 (1.06–1.32)	**0.003**
	≥100 and ≤200 Cells/mL^3^	1293	Ref		Ref	
	<100 Cells/mL^3^	1464	1.00 (0.92–1.09)	0.98	0.98 (0.89–1.08)	0.63
**First Line ART Regimen Prescribed**	TDF+3TC+NVP or EFV	671	0.61 (0.54–0.69)	**<0.0001**	0.73 (0.62–0.84)	**<0.0001**
	ZDV+3TC+NVP or EFV	2942	0.88 (0.83–0.94)	**<0.0001**	0.96 (0.88–1.05)	0.37
	d4T+3TC+NVP or EFV	916	Ref		Ref	
**Total Time on ART**	>15 and ≤20 months	515	1.54 (1.38–1.72)	**<0.0001**	1.35 (1.15–1.58)	**0.0002**
	>12 and ≤15 months	880	1.33 (1.20–1.48)	**<0.0001**	1.20 (1.05–1.37)	**0.007**
	>9 and ≤12 months	800	1.30 (1.17–1.45)	**<0.0001**	1.22 (1.07–1.39)	**0.003**
	>6 and ≤9 months	1025	1.20 (1.08–1.34)	**0.001**	1.20 (1.06–1.36)	**0.005**
	≥3 and ≤6 months	1309	Ref		Ref	

a Totals do not always sum to 4,529 due to missing data;

b A total of 3,136 subjects included in the adjusted model; OR, odds ratio; CI, confidence interval.

In the multivariate GEE regression which included 3,135 (70.4%) patients with complete data and adjusting for ART duration, decreased odds of Rx<95% remained associated with TDF-containing regimen (OR = 0.73) and disclosure of HIV status to spouse or family member (OR = 0.85). Compared to patients with CD4 cell counts between 100 and 200, patients with CD4 above 200 were at increased odds of Rx<95%; OR = 1.18 (95%CI: 1.06–1.32) for patients with CD4 cell counts between 200 and 350 and OR = 1.25 (95%CI: 1.05–1.49) for patients with CD4 cell counts greater than 350. There was no difference in Rx<95% between patients with CD4<100 and those with CD4 between 100 and 200 cells/mm^3^ (OR = 0.98, p = 0.66). Longer travel time to the clinic (>2 hours) was associated with increased odds of Rx<95% (OR = 1.11; 95%CI: 1.01–1.23). Increased odds of Rx<95% was also associated with increasing time on ART.

**Figure 2:**
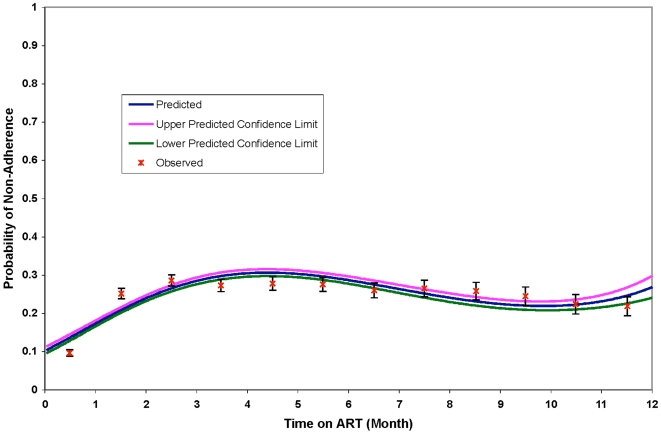
Modeled Probability of Non-adherence within the First 12 Months of ART Initiation.

In the regression model where time since ART initiation was modeled as a continuous risk factor, the best fitting model included a cubic polynomial model for time. Modeled probabilities of Rx<95% across each time-points and 95% confidence intervals for the final best-fitting model are shown in **Figure 2**. The risk of non-adherence was highest in the first 6 months after ART initiation with the proportion of patients having Rx<95% that ranges from 28% at 3 months to 26% at 6 months.

## Discussion

In a large antiretroviral therapy program in Nigeria, approximately 1 in 4 patients were LTFU between March 2005 and July 2006; this is very similar to findings from other HIV treatment programs in sub-Saharan Africa[2], [4]. In our analysis, increased risk of LTFU was associated with being male, younger age, having CD4 level at ART initiation below 100, and treatment with a d4T-containing first-line regimen. These risk associations did not differ between patients who discontinued care after one visit and patients who returned after one visit but were eventually lost. Increased risk of non-adherence was associated with younger age, having CD4 level at ART initiation below 200, and long travel time to the clinic while better adherence was associated with disclosure of HIV status to family members and first-line treatment with TDF-containing regimen.

The finding that a higher proportion of women initiated on ART remained in follow-up care in this cohort mirrors findings from other treatment cohorts in Africa[4] and may reflect gender-differences in health-seeking behaviors which has been shown to affect retention in care in other resource-limited countries[24], [25]. For HIV treatment programs to be effective in optimizing retention, strategies are needed to effectively engage and support men receiving ART care. Behaviors more common among men which may impact LTFU, such as alcohol use, were not assessed in this study.

Although the majority of the patients had a CD4 level within the range of requiring ART according to the National and WHO guidelines[26], higher LTFU was observed for both patients whose CD4 level was low (less than 100) and whose CD4 level was high (above 350). The bimodal relationship suggests that the risk associated with higher CD4 group may be due to patients who considered themselves or felt “healthy” and need not return for care while the risk associated with lower CD4 is suggestive of patients who were too sick to engage and continue on care, or who may have died. Significant attrition risk in the first 6 months of treatment can be attributable to death[27], [28], [29], [30]. Incomplete tracking of mortality is a major challenge in the context of ART scale-up in settings such as this.

Nearly half of patients LTFU never returned after receiving their first prescription of ARVs. This figure is much larger than previously reported across different ART programs in Africa[28]. Comparing these “early” LTFU to patients who were subsequently lost showed that they were more likely to have CD4 below 100. While no other differences were observed, physicians in our program do preferentially placed sicker patients on a TDF-containing regimen. These data are based upon very early program implementation. At a time of ambitious treatment goals for PEPFAR, it is likely that inadequate resources and training were focused on treatment preparation, and implementation of ART services stressed a weak health service delivery system. Better treatment preparation was implemented in response to these findings. While there is now a national policy of free ART services at public facilities in Nigeria, there was not a national policy at the time regarding institutional patient charges for ART services. There was not a national ART registration system which would detect the same patient receiving services at multiple care sites. There were alternative care sites with different charging policies near the study sites at the time. Thus, some patient migration to other ART care sites cannot be excluded.

In the analysis of the impact of various first-line regimens, patients initiated on a stavudine-containing first-line regimen had higher risk of LTFU compared to other regimens containing either zidovudine or tenofovir. Stavudine has been shown to be associated with peripheral neuropathy[31], [32], lactic acidiosis[33], [34], and lipoatrophy[35], [36], and other metabolic complications[37] Although data on side effects of ART medications were infrequently recorded or reported by patients in this program, other studies have found that side effects can be associated with LTFU[6]. Stavudine-containing regimens are no longer recommended in resource-rich countries because of toxicity and side effects but are still widely used in Nigeria (∼20% of all first-line regimens prescribed in 2008 and 2009) and other resource-limited countries because of availability supplied by donors. While we did not observe a secular trend in regimen choices of physicians or differences in regimen choice by site, it is possible that health care providers who were more likely to prescribe stavudine may also be less competent with patient education and adherence support. Additional studies are needed to evaluate the long-term patient-level effects and programmatic outcomes related to various first line ART options.

At the end of the analysis time, more than one third of the cohort had an overall Rx<95%. There was a concordance between the proportion of patients with at least one visit interval with Rx<95% (i.e. an episodic Rx<95%) and the proportion of patients with an overall summary Rx<95%, suggesting that once patients demonstrate poor adherence in the course of follow-up, the behavior is likely to continue and intensive adherence support should be initiated immediately for this group of patients. The vast majority of episodes of adherence <95% were simply due to the patient not returning to the health care facility for pharmacy refill in a timely matter. Periodic discontinuation (≥48 hours) of a non-nucleoside-based ART regimen is particularly concerning for the development of resistance due to the long half-life of these agents[38]. The risk of non-adherence observed to be highest in the first 6 months after ART initiation suggests that limited adherence support resources should be focused on patients early in their treatment course.

Good adherence was associated with disclosure of HIV status to either spouse or family member and with a tenofovir-containing first-line regimen. The protective effect of disclosure on non-adherence is the first finding from a large treatment program in Nigeria and speaks to the importance of stigma as a barrier to effective life-long treatment adherence as also reported from South Africa[10], Uganda[39], Botswana[40], and Zambia [41]. Because disclosure to family members is imperative to ensure their support, identifying tools or resources that can minimize the possible risks and maximize the potential benefits of disclosure should be useful in improving the care of persons living with HIV/AIDS. In addition to counseling on disclosure, engaging the community in HIV/AIDS care could potentially create an environment that reduces stigma and encourages disclosure to family members[42].

While our program did not collect information on religion, interestingly, patients with Q'uranic education were at increased risk of being non-adherent. Studies on understanding the cultural context of health care maintenance and adapting HIV treatment and care services to local customs are important for ensuring optimal clinical outcomes in various patient populations. This finding also underscores the importance of religious organizations and community leaders in further supporting people living with HIV/AIDS.

Patients who spent considerable time traveling to the treatment facilities were at increased risk of non-adherence. Although the number of treatment facilities in Nigeria continue to increase, patients may continue avoid accessing care from facilities within their communities because of stigma. As a result, scale up of treatment facilities must be coupled with support from the communities. Beyond the standard and simple public health approach to ART and based on the data presented herein, the ACTION project has initiated a treatment support structure with emphasis on multiple visit pre-treatment preparation, promotion of disclosure, and treatment companions. The ACTION project is also implementing different models of comprehensive care delivery such as task-shifting, expanding development of ART points of service at the local primary health center level, home-based care[43], treatment companions[44], more extensive and targeted counseling, and frequent visits and monitoring by health care workers[45], [46] in order to provide structural support for adherence and retention.

Our findings should be interpreted in light of several caveats. First, the retrieved data were based on a clinical cohort rather than a “classical” structured cohort that resulted in a variable interval of follow-up time for patients. We accounted for these variations in the analysis using the GEE approach[20]. Additional analyses will be necessary to examine the influence of these risk factors beyond the first 12 months of ART initiation. Second, the data on side effects and mortality, which could further help explain interesting findings, were incomplete because of logistical challenges in this setting. Third, because viral load measurements were not routinely performed, we were not able to characterize patients' virologic response to ART. Lastly, pharmacy refill records were used as a proxy for level of adherence to ART because this was deemed to be the most objective approach for retrospective analysis in this cohort. Structured adherence self-report measures were utilized as a part of routine care, but we found that many patients with poor pharmacy refill rates self-reported excellent adherence[47]. Although pharmacy refill data does not measure medication-taking behavior, it is considered to be a more objective measure compared to patient-report[48]. Moreover, given the strong correlation between adherence and immunologic response reported in other studies[49] coupled with limited resources for obtaining viral load measurements, pharmacy refill data is a convenient tool for determining whether a patient is likely to fail therapy in the absence of viral load measurements. These limitations may be considered significant but they do reflect the realities of HIV treatment programs in many resource-limited settings.

In summary, although rapid scale-up of ART in Nigeria has been remarkable, issues such as patient retention and adherence are likely to remain critical factors in patient-level and program-level success. A variety of models of service delivery such as availability of ART services at the primary health center level, home-based care, and task-shifting are being piloted and evaluated to enhance treatment adherence support. Our findings formed the basis for implementing multiple pre-treatment visit preparation that promote disclosure and active community outreaching to support retention and adherence. Keeping patients on treatment should be considered as important a factor as boosting the numbers of patients initiation ART. Coupled with expansion of treatment access points of care to communities to diminish travel and to create an environment that reduces stigma may further have a positive impact on adherence.
